# Replicative Senescence of Mesenchymal Stem Cells: A Continuous and Organized Process

**DOI:** 10.1371/journal.pone.0002213

**Published:** 2008-05-21

**Authors:** Wolfgang Wagner, Patrick Horn, Mirco Castoldi, Anke Diehlmann, Simone Bork, Rainer Saffrich, Vladimir Benes, Jonathon Blake, Stefan Pfister, Volker Eckstein, Anthony D. Ho

**Affiliations:** 1 Department of Medicine V, University of Heidelberg, Heidelberg, Germany; 2 Department of Physiology and Pathophysiology, University of Heidelberg, Heidelberg, Germany; 3 Genomics Core Facility, European Molecular Biology Laboratory, Heidelberg, Germany; 4 Department of Pediatric Oncology, Hematology and Immunology, University of Heidelberg, Heidelberg, Germany; 5 Heidelberg Academy of Sciences and Humanities, Heidelberg, Germany; Baylor College of Medicine, United States of America

## Abstract

Mesenchymal stem cells (MSC) comprise a promising tool for cellular therapy. These cells are usually culture expanded prior to their application. However, a precise molecular definition of MSC and the sequel of long-term *in vitro* culture are yet unknown. In this study, we have addressed the impact of replicative senescence on human MSC preparations. Within 43 to 77 days of cultivation (7 to 12 passages), MSC demonstrated morphological abnormalities, enlargement, attenuated expression of specific surface markers, and ultimately proliferation arrest. Adipogenic differentiation potential decreased whereas the propensity for osteogenic differentiation increased. mRNA expression profiling revealed a consistent pattern of alterations in the global gene expression signature of MSC at different passages. These changes are not restricted to later passages, but are continuously acquired with increasing passages. Genes involved in cell cycle, DNA replication and DNA repair are significantly down-regulated in late passages. Genes from chromosome 4q21 were over-represented among differentially regulated transcripts. Differential expression of 10 genes has been verified in independent donor samples as well as in MSC that were isolated under different culture conditions. Furthermore, miRNA expression profiling revealed an up-regulation of hsa-mir-371, hsa-mir-369-5P, hsa-mir-29c, hsa-mir-499 and hsa-let-7f upon *in vitro* propagation. Our studies indicate that replicative senescence of MSC preparations is a continuous process starting from the first passage onwards. This process includes far reaching alterations in phenotype, differentiation potential, global gene expression patterns, and miRNA profiles that need to be considered for therapeutic application of MSC preparations.

## Introduction

Mesenchymal stem cells (MSC) represent a multipotent adult stem cell population that, given the appropriate culture conditions, is able to differentiate into different mesodermal cell lineages including osteocytes, chondrocytes, and adipocytes. Albeit controversial, there is evidence that MSC can also differentiate into myocytes and cardiomyocytes and even into cells of non-mesodermal origin including hepatocytes and neurons [1]–[4]. Reliable markers for the definition of the multipotent fraction have not yet been defined and hence these cells have alternatively been named mesenchymal stromal cells [5]. Due to the lack of reliable molecular markers, MSC are concurrently defined by: a) plastic adherent growth, b) immunophenotype and c) their *in vitro* differentiation potential [6], [7]. Nevertheless, human MSC raise high hopes in various therapeutic applications and their use is concurrently tested in various clinical trials [7].

MSC have a limited lifespan *in vitro* as any normal, somatic cell. After a certain number of cell divisions, MSC enter senescence, which is morphologically characterized by enlarged and irregular cell shapes and ultimately a stop of proliferation. This phenomenon was first described in the 1960s by Leonard Hayflick [8]. Since then, it is debated if the so-called “Hayflick limit” might reflect the aging process of the whole organism. If cellular senescence triggers aging, this would be of utmost importance for all adult stem cells. The tissues of any organism are continuously renewed by adult stem cells and impairment of their function would inevitably result in aging [9]. Recent studies have indicated that murine and human MSC exhibit reduced differentiation potential upon prolonged *in vitro* culture [10]–[13]. Furthermore, senescence of MSC might limit their therapeutic applications. Thus, analysis of *in vitro* senescence in MSC is crucial for basic research as well as for quality control in cellular therapy.

The molecular mechanisms that underlie senescence are still poorly understood. Two fundamental ways have been hypothesized how this process may be governed: replicative senescence might either be the result of a purposeful program driven by genes or rather be evoked by stochastic or random, accidental events [14]. Most likely, it is an interplay of both mechanisms that promotes aging at various levels. Progressive shortening of the telomeres or modified telomeric structure has been associated with replicative senescence although this mechanism is unlikely to be the only cause of this phenomenon [15], [16]. There is also evidence that senescence involves DNA damage, accumulation of the cyclin-dependent kinase inhibitor p16INK4a and oxidative stress [9], [17], [18]. Clearly, cellular senescence is a complex process and the sequence of its molecular events is thus far unknown. Additionally, it is not known how senescence influences the overall expression of coding genes and micro RNAs in MSC.

With this in mind, we devised a study to analyze how morphology, immunophenotype and differentiation capacity of MSC is affected by *in vitro* expansion. In parallel, we analyzed how mRNA and miRNA expression profiles change upon culturing and *in vitro* propagation. We intended to gain insight into the molecular effects of replicative senescence even at early passages that would have impact for the quality control of MSC preparations used for therapeutic application.

## Results

### Long-term growth kinetics and morphology of MSC

**Figure 1:**
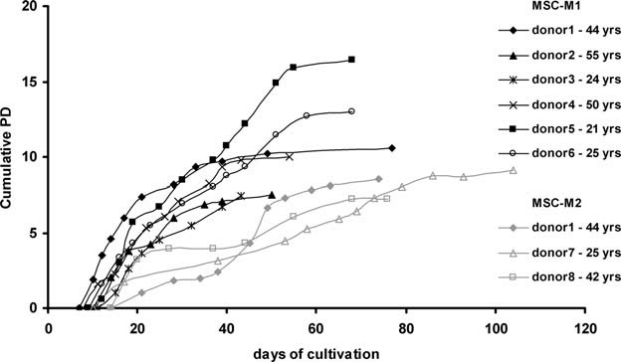
Long-term growth curves. Long-term growth curves are demonstrated for six MSC preparations isolated under culture conditions M1 and for three MSC preparations isolated under culture conditions M2. Cell numbers were determined at the end of every passage and cumulative population doublings (PD) were calculated in relation to the cell numbers at the first passage. The age of each donor is provided.

**Figure 2:**
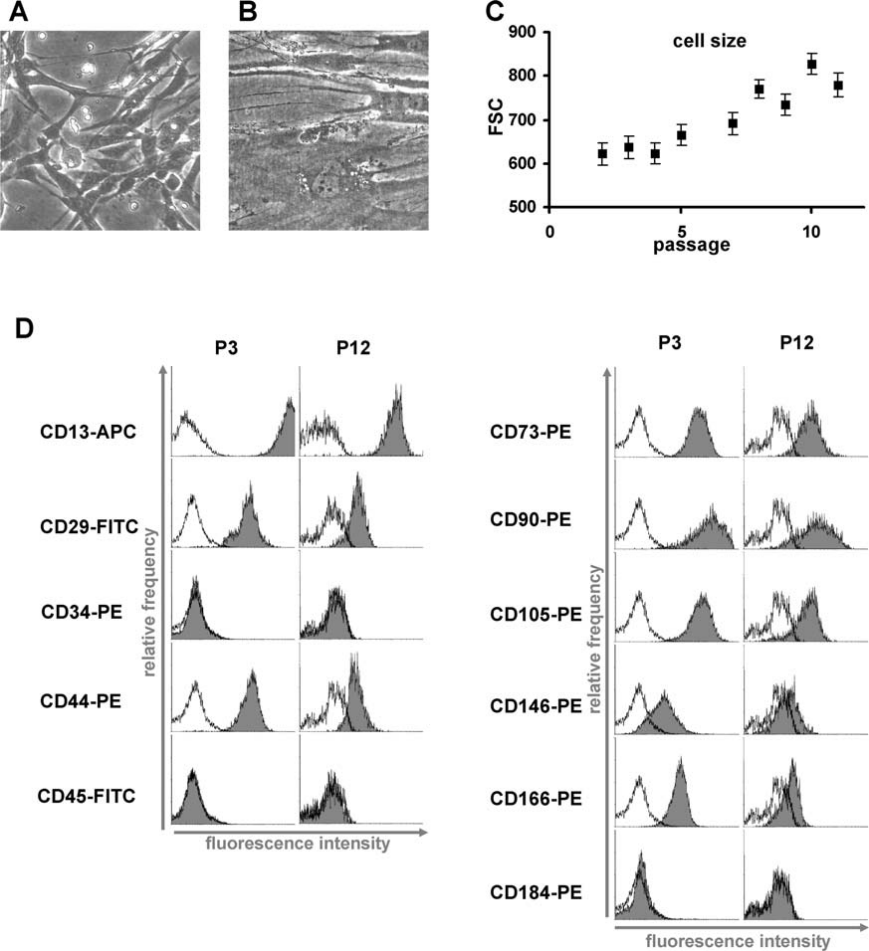
Morphologic changes and immunophenotype upon senescence. Replicative senescence is reflected by dramatic changes in morphology. Cells enlarge, generate more vacuoles and cellular debris and ultimately stop proliferation. Representative morphology of MSC in early (P3) and senescent passage (P12) is presented (A, B). The continuous increase in cell size and granularity is reflected by the increasing forward-scatter signal in flow cytometry (FSC, ±SD; C). Immunophenotypic analysis of all MSC preparations was in accordance with the literature whereby the detection level for positive markers was much higher in early passages compared to late passages (black line  =  autofluorescence; D). A representative analysis of three preparations is demonstrated.

Plastic-adherent fibroblast-like colonies were observed in all donor samples within the first days of cultivation. Proliferation gradually decreased in all samples in the course of long-term cultivation until the cells finally stopped to proliferate. Under culture conditions with low serum content and supplemented growth factors (MSC^M1^) cells proliferated relatively fast for 43 to 77 days, whereas MSC cultured in the commercial cambrex medium with 10% serum (MSC^M2^) resulted in prolongated growth for 74 to 104 days. Long-term growth curves differed considerably between the eight donor samples and the proliferation rate varied over the time course although the cells were always passaged at the same density of about 70% confluence (figure 1). The cumulative number of population doublings varied between 6 and 16 plus an estimated 7 to 9 population doublings during the initial colony formation. Overall, this would result in a maximal cell number of 3.6×10^8^ to 7.1×10^11^ for MSC^M1^ and 1.1×10^8^ to 1.7×10^8^ for MSC^M2^. Replicative senescence led to previously observed typical morphological changes (figure 2A, B): cells became much larger with irregular and flat shape, and nuclei became more circumscribed in phase contrast microscopy. The cytoplasm began to be granular with many inclusions and aggradations appearing to be cell debris increased. Morphologic changes were not restricted to senescent stages but represented continuous alterations in the course of long-term culture of MSC.

### Immunophenotype

Immunophenotypic analysis represents one of the major parameters for the characterization of MSC preparations. Here, we analyzed the impact of senescence on the immunophenotype of MSC^M1^ preparations by flow cytometry. The median forward scatter signal increased during *in vitro* cultivation and this may be attributed to the continuously increasing cell size and granularity (figure 2C). A panel of 11 surface markers was tested (figure 2D). All MSC preparations were negative for the hematopoietic markers CD34 and CD45 and positive for CD13, CD29, CD44, CD73, CD90, CD105, CD146, and CD166 as expected [5], [19], [20]. Using this panel of markers, flow cytometry was not able to discern any distinct characteristics with regard to the heterogeneous composition of MSC subpopulations. Surprisingly, the level of surface antigen detection was much higher in early passages when compared to senescent passages. At the same time autofluorescence of MSC increased and this may be due to accumulation of highly fluorescent lipofuscin at later passages. These results indicate that the composition and expression level of surface markers varies upon long-term expansion.

### *In vitro* differentiation

**Figure 3:**
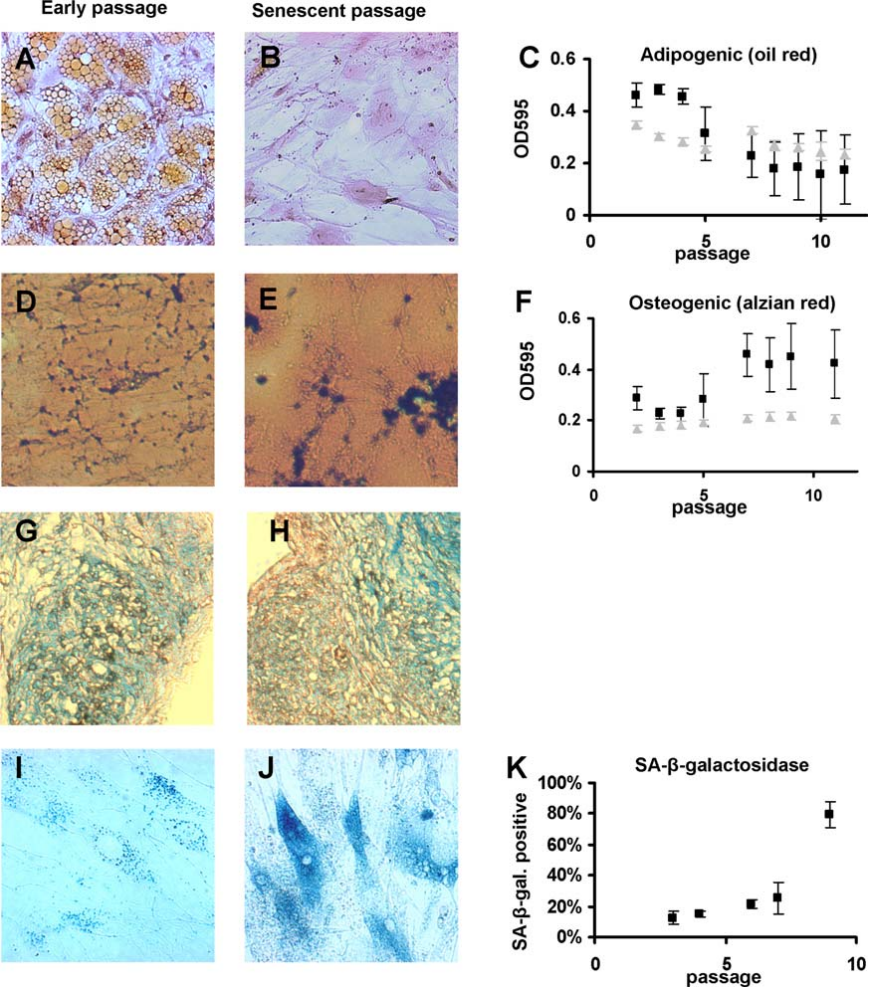
*In vitro* differentiation. MSC of different passages were simultaneously differentiated along adipogenic or osteogenic line. Fat accumulation was visualized by Oil Red-O staining. Adipogenic differentiation potential decreased in higher passages (A, B, C). In negative controls without differentiation (grey triangles) no fat accumulation was observed but the cells grew to a higher density which also resulted in higher OD. Osteogenic differentiation was visualized by van Kossa staining (not demonstrated) or Alizarin red staining. There was a higher propensity for osteogenic differentiation in higher cell passages (D, E, F). Senescence associated β-galactosidase staining increases in the later passages (G, H, I). Representative results of three independent MSC preparations are demonstrated (±SD).

Another key parameter for defining MSC is their potential to differentiate along the adipogenic, osteogenic and chondrogenic lineages [1], [21]–[23]. Here, we have analyzed how the *in vitro* differentiation potential is affected by replicative senescence in MSC^M1^ (figure 3). The potential for adipogenic differentiation was confirmed following standard protocols, and lipid vesicles were stained by Oil red O. Fat formation was much more effective in early MSC passages indicating that adipogenic differentiation potential decreases in the course of *in vitro* senescence. In contrast, the propensity for osteogenic differentiation increased in later passages as demonstrated upon staining with either von Kossa or Alizarin red dye. Chondrogenic differentiation could also be induced in our MSC. However, despite the same initial cell number the pellets from early passages were larger and denser than those from later passages and this prevented a reliable semi-quantitative comparison of glycosaminoglycan expression. These results support the notion that long-term culture has an incremental impact on differentiation potential of MSC.

### Molecular markers for senescence

Given the functional implications of *in vitro* senescence on MSC, we were interested in the analysis of senescence biomarkers. Because the enzyme lysosomal pH6 β-galactosidase (SA-β-gal) has been shown to be active in senescent human fibroblasts, but not in quiescent, pre-senescent or differentiated cells [24], we have employed SA-β-gal as senescence marker. In the last passages of MSC^M1^ the percentage of SA-β-gal positive cells as well as the intensity of the staining increased (figure 3). However, a more specific molecular marker would be necessary to grade the level of senescence of MSC preparations.

### Differential gene expression upon replicative senescence

**Figure 4:**
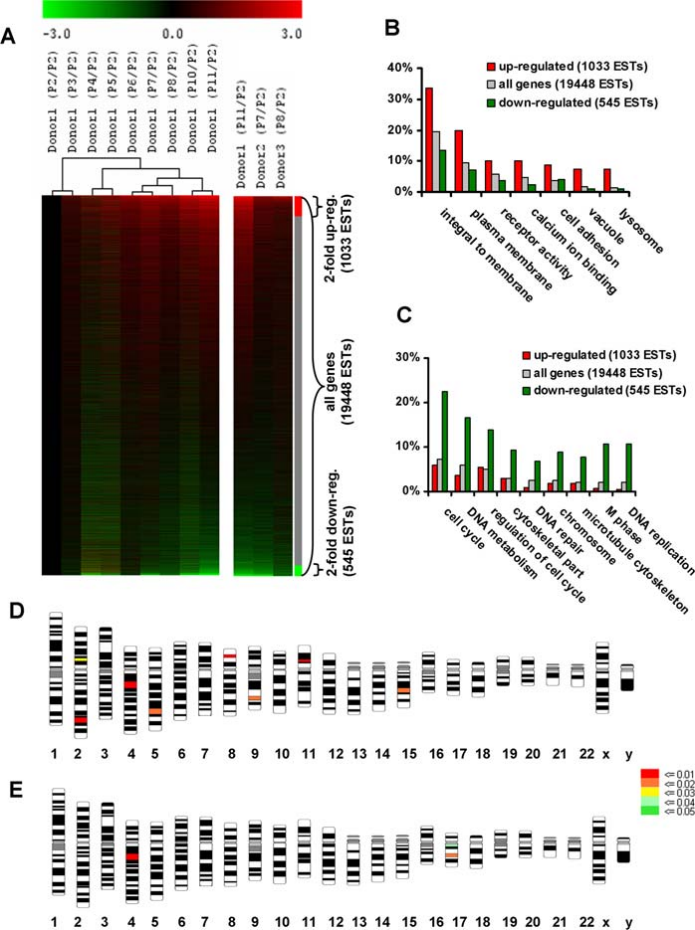
mRNA expression profile of MSC changes extensively with higher passage. Differential gene expression of senescent passages *versus* P2 was analyzed by Affymetix GeneChip technology in three independent MSC preparations. 19,448 ESTs that were detected as present in at least 10 of 13 hybridizations were ordered according to their log_2_ratio. 1033 ESTs were more than 2-fold up-regulated (red) and 545 were more than 2-fold down-regulated (green). Analysis of different passages of donor1 demonstrated increasing changes in the global gene expression pattern during *in vitro* senescence (A). GeneOnthology analysis was performed for the subsets of genes that were >2-fold up-regulated or >2-fold down-regulated in comparison to all genes detected as present on the microarray. The percentages of genes that contributed to representative categories are depicted (B,C; P<0.0001). Probabilities of co-localization of regulated genes plotted onto a human karyogram. The probability of representation of 2-fold up-regulated genes (D) and 2-fold down-regulated genes (E) on chromosomal regions is indicated by color coding.

To determine mRNA expression changes indicative for *in vitro* senescence of MSC^M1^, we analyzed mRNA expression patterns of corresponding early passages (P2) and senescent passages (PX) of the same donor. We identified global changes in mRNA expression profiles that were commonly observed in three different donors (figure 4). The most significant differentially expressed genes are summarized in table 1 (SAM analysis, FDR<3).

**Table 1:** Differentially expressed mRNA upon *in vitro* senescence.

Genname	Short Cut	Affymetrix ID	Log_2_ratio	SD
**Up-regulated in senescent passages**				
glycoprotein (transmembrane) nmb	GPNMB	201141_at	4.17	1.01
Regeneration-associated muscle protease homolog	RAMP	213661_at	3.94	1.03
mannosidase, alpha, class 1C, member 1	MAN1C1	218918_at	3.77	1.17
glucosaminyl (N-acetyl) transferase 3, mucin type	GCNT3	219508_at	3.71	1.05
glycoprotein (transmembrane) nmb	GPNMB	1554018_at	3.39	0.79
secretogranin II (chromogranin C)	SCG2	204035_at	3.33	1.04
hypothetical protein LOC119548	LOC119548	1558846_at	3.08	0.62
PERP, TP53 apoptosis effector	PERP	217744_s_at	2.83	0.30
mucolipin 3	MCOLN3	229797_at	2.66	0.48
ectonucleotide pyrophosphatase/phosphodiesterase 5	ENPP5	227803_at	2.64	0.76
cathepsin K (pycnodysostosis)	CTSK	202450_s_at	2.64	0.35
activating transcription factor 3	ATF3	202672_s_at	2.35	0.53
leucine-rich repeat-containing G protein-coupled receptor 7	LGR7	231804_at	2.28	0.10
disabled homolog 2, mitogen-responsive phosphoprotein	DAB2	240873_x_at	2.27	0.31
solute carrier family 11, member 2	SLC11A2	203124_s_at	2.08	0.34
Homo sapiens transcribed sequences		229308_at	2.06	0.47
lymphocyte antigen 96	LY96	206584_at	2.04	0.43
DnaJ (Hsp40) homolog, subfamily B, member 4	DNAJB4	203811_s_at	2.00	0.30
solute carrier family 16, member 6	SLC16A6	230748_at	1.89	0.30
solute carrier family 11, member 2	SLC11A2	203123_s_at	1.88	0.06
signal transducer and activator of transcription 1	STAT1	209969_s_at	1.75	0.33
hypothetical protein dJ462O23.2	DJ462O23.2	214579_at	1.74	0.14
GM2 ganglioside activator protein	GM2A	1559776_at	1.71	0.33
signal transducer and activator of transcription 1	STAT1	M97935_MA_at	1.71	0.26
interferon-induced protein with tetratricopeptide repeats 1	IFIT1	203153_at	1.68	0.13
hypothetical protein FLJ31715	FLJ31715	1553775_at	1.53	0.12
clone IMAGE:3632546	LOC643988	227185_at	1.50	0.10
carbonic anhydrase XI	CA11	209726_at	1.48	0.18
prion protein (p27-30)	PRNP	215707_s_at	1.37	0.15
SEC14-like 4 (S. cerevisiae)	SEC14L4	239492_at	1.31	0.11
chromosome 20 open reading frame 22	C20orf22	228123_s_at	1.28	0.10
glutaredoxin (thioltransferase)	GLRX	209276_s_at	1.23	0.09
**Down-regulated in senescent passages**				
chemokine (C-X-C motif) ligand 6	CXCL6	206336_at	−2.09	0.23
hyaluronan synthase 1	HAS1	207316_at	−2.40	0.22
retinoic acid receptor responder (tazarotene induced) 1	RARRES1	221872_at	−4.31	0.82
tumor necrosis factor (ligand) superfamily, member 11	TNFSF11	210643_at	−6.10	1.46

36 Genes that were significantly differentially expressed between early and senescent passage of three independent donor samples (SAM, FDR = 3).

Up-regulated genes included: human glycoprotein NMB (GPNMB) that has high homology to osteoactivin, a glycoprotein that plays a role in osteoblast differentiation and function [25]; regeneration-associated muscle protease homolog (RAMP) that might play a role in regeneration of skeletal muscle; p53 apoptosis effector related to PMP-22 (PERP) that plays a role in stratified epithelial integrity and cell–cell adhesion by promoting desmosome assembly and acts as an effector for the p53-dependent apoptotic pathway [26]; Lymphocyte antigen 96 (LY96) that enhances TLR4-dependent activation of NF-kappa-B; Signal transducer and activator of transcription 1 (STAT1) involved in the regulation of Interferon γ-activated sequences; and the prion protein (PRNP), which has been implicated in various types of transmissible neurodegenerative spongiform encephalopathies that such as Creutzfeldt-Jakob disease which usually manifest at higher ages [27]. Furthermore, the senescence associated markers cyclin-dependent kinase inhibitor 2A (p16; Affymetrix ID: 207039_at; log_2_ratio = 2.12±0.95) and plasminogen activator inhibitor type 1 (PAI; Affymetrix ID: 202627_s_at; log_2_ratio = 2.14±1.74) were up-regulated but they did not reach the level of significance. Genes that were down-regulated during replicative senescence included: hyaluronic acid synthetase 1 (HAS1) that mediates expression of the corresponding unbranched polysaccharide which represents an important constituent of the extra cellular matrix; inhibitor of DNA binding 1 (ID1; Affymetrix ID: 208937_s_at; log_2_ratio = −3.58±0.92) that is higher expressed in MSC preparations in comparison to non-multipotent fibroblasts [20]; and osteoprotegerin ligand (TNFSF11) that has been suggested as an osteoclast differentiation and activation factor [28].

Differential expression of 10 selected genes was verified by quantitative RT-PCR and the results were always in accordance with the microarray data in either the same three MSC preparations or in three additional independent donor samples. Furthermore, differential gene expression was also verified for nine of these genes in three MSC preparations that were isolated under different culture conditions (MSC^M2^). Thus, the identified changes in the global gene expression profile are highly consistent in different MSC preparations.

Our analysis revealed 1033 transcripts with a more than 2-fold up-regulation in senescent cells whereas 545 transcripts were more than 2-fold down-regulated. GeneOntology (GO) analysis demonstrated that up-regulated genes in senescent cells were highly overrepresented (P<0.0001) in the categories integral to membrane (GO:16021), plasma membrane (GO: 5886), receptor activity (GO:4872), cell adhesion (GO:7155), vacuole (GO:5773) and lysosome (GO:5764) (figure 4B). These findings are in line with the observed enlargement of the membrane compartment and vacuole formation in higher passages. Less abundant genes in senescent passages were associated with cell cycle (GO:7049), DNA metabolism (GO:6259), regulation of cell cycle (GO:51726), cytoskeletal part (GO:44430), DNA repair (GO:6281), chromosome (GO:5694), microtubule cytoskeleton (GO:15630), M-phase (GO:279) and DNA-replication (GO:6260) (figure 4C). These categories are perfectly in line with the reduced proliferation potential, accumulation of DNA defects and changes in cellular cytoskeleton as reflected by the senescence phenotype that was observed morphologically.

All data were probed for evidence of physical clustering among the senescence associated genes. Genes that were 2-fold up-regulated in senescent cells were significantly over-represented on chromosome bands 4q21, 11p13, 2q33, 5q14 and 8p22; whereas genes that were 2-fold down-regulated were over-represented on 4q21, 10q21.1, 5q12, 10q23.33, 1p31.2, 3q25.32 and 8q23 (P<0.01). Interestingly, the most significant over-representation (P<0.0005) for up- and down-regulated genes are co-localized at the same hot-spot on chromosome 4q21 (figure 4D, E).

**Figure 5:**
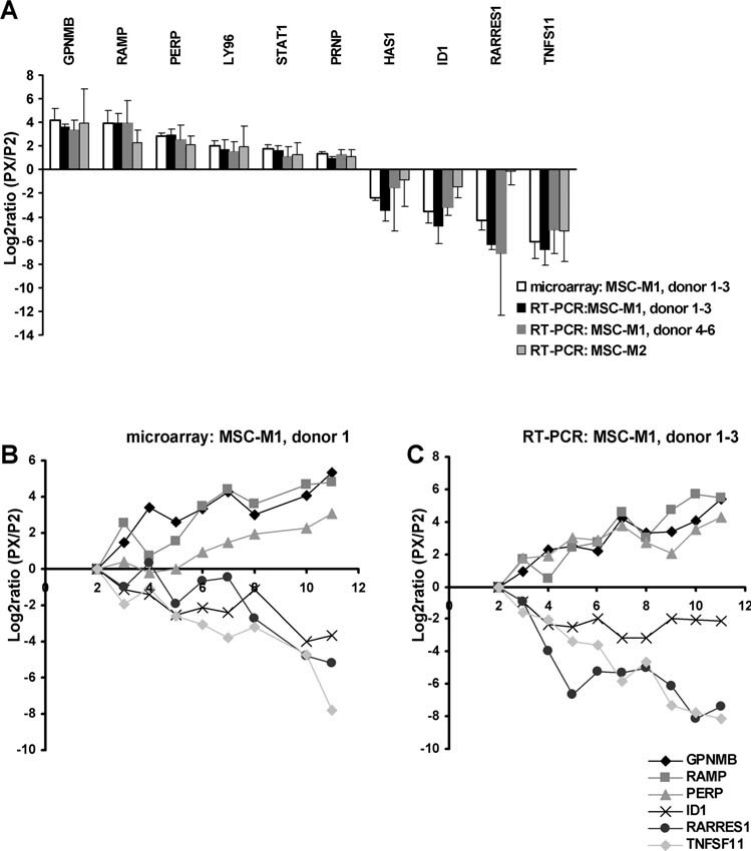
QRT-PCR validation of mRNA expression. Differential expression of senescent passage (PX) *versus* P2 was validated by using QRT-PCR for 10 genes (A). Results were in line with microarray data for all tested genes, investigating either the same three MSC preparations (donor 1–3) or three independent donor samples that were isolated in the same culture medium M1 (donor 4–6). Furthermore, differential gene expression was also observed in three MSC preparations isolated under different culture conditions (M2). Differential mRNA expression was not restricted to senescent passages but increased during the course of replicative senescence (B,C).

The question remained, if these changes in mRNA expression are restricted to senescent passages, or if they represent incremental modifications in the course of cellular aging. Thus, we analyzed different cell passages of the same MSC preparation by microarray analysis. We observed continuous changes in global gene expression patterns as reflected by unsupervised hierarchical cluster analysis. Variation in the gene expression profile was already observed in very early passages, when morphological differences were not obvious. This could also be confirmed by QRT-PCR in samples from additional donors (figure 5). Thus, many changes in gene expression patterns of MSC preparations are not restricted to senescent passages, but are increasingly acquired upon *in vitro* expansion.

### Differential microRNA expression upon replicative senescence

**Figure 6:**
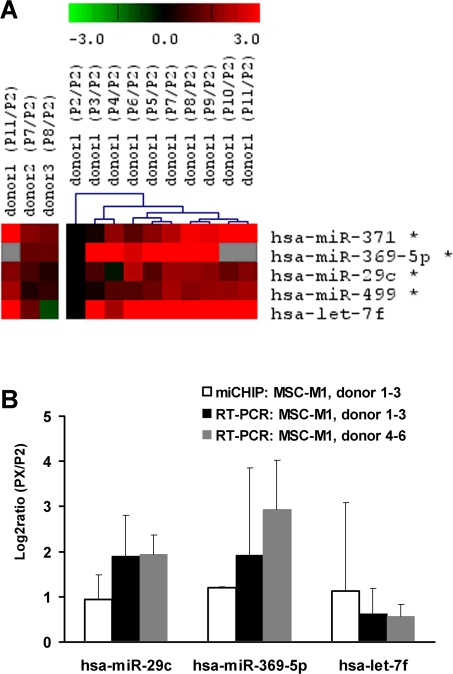
miRNA expression changes upon *in vitro* senescence. miRNA expression in early and senescent passages of three MSC preparations was determined by microarray analysis (miCHIP) [29]. Five miRNAs that are up-regulated during senescence are depicted (*  =  significant by SAM analysis). miRNA expression was also analyzed in the sequential passages of donor 1 and hierarchical cluster analysis revealed that expression of these miRNAs was overall increased during senescence (A). Furthermore, differential miRNA expression was validated by QRT-PCR for hsa-mir-29c, hsa-mir-369-5p and hsa-let-7f in the three MSC preparations that were used for microarray analysis as well as in three additional samples (B).

Senescence might be associated with the differential expression of miRNAs. We have compared miRNA profiles of early *versus* senescent MSC^M1^ passages using a microarray platform based on locked nucleic acids (miCHIP) [29]. Data analysis, normalization and statistical methods were performed described for the mRNA expression analysis. SAM analysis identified a group of five significantly up-regulated miRNAs, (FDR<1): hsa-mir-371, hsa-mir-369-5P, hsa-mir-29c, hsa-mir-499 and hsa-mir-217 (signal intensity of hsa-mir-217 was very low and thus not considered for subsequent analysis). In addition, miRNA expression was analyzed in the different passages of donor 1 and hierarchical cluster analysis indicated that expression of these miRNAs increases in the course of replicative senescence. Differential expression of three miRNAs (hsa-mir-369-5P, hsa-mir-29c and let-7f) was validated by using quantitative RT-PCR in all three donor samples as well as in three independent donor samples (figure 6). QRT-PCR results were in line with miCHIP analysis indicating that the expression of these miRNAs is up-regulated in the course of replicative senescence of MSC.

## Discussion

MSC have paved their way towards therapeutic application although there is little knowledge about specific molecular markers for this population and the impact of culture expansion methods. In this study, we demonstrate that replicative senescence of MSC has functional implications on surface marker expression and differentiation potential and that it evokes consistent changes in the global gene expression and miRNA expression profiles of MSCs from different donors. These senescence-associated effects were not restricted to senescent passages, but are continuously acquired from the onset of *in vitro* culture.

Many studies have reported that MSC undergo the typical Hayflick phenomenon of cellular senescence with decreasing proliferation and changes in cell morphology. We have found that senescence occurred after a cumulative number of population doublings ranging from 6 to 16 population doublings. In addition, an estimated 7 to 9 population doublings took place during the initial colony formation. Thus, the total number of population doublings would be between 13 and 25 and this is compatible with expansion rates of 10^4^-fold to 10^8^-fold. This is in line with observations of other groups [10], [30]. The pace of senescence might be affected by the culture conditions. Colter et al. reported that single cell derived MSC clones could be expanded up to 50 population doublings in about 10 weeks if cultured by repeated passage at low density whereas cells stopped growing after 15 passages if passed at high cell density [31]. Other authors suggested that lower oxygen concentrations could enhance the maximal number of population doublings [32]. MSC isolated from different tissues have different functional properties including different long-term growth kinetics [30], [33]. Furthermore, there is evidence for a negative correlation between donor age and the proliferative capacity of MSC although this is still under debate [10], [34]–[36]. Data of this study might demonstrate a tendency for higher cumulative population doublings in MSC form younger donors but more probes would be necessary to verify this effect.

In this study, we demonstrated that *in vitro* expansion has a major impact on a) the morphology of plastic adherent growth, b) the level of surface marker expression and c) adipogenic and osteogenic differentiation potential and thus on all parameters that are concurrently used for the definition of MSC [6]. Other authors did not find differences in surface marker expression of senescent MSC [10], [36]. This might be due to the fact that in these studies, the level of surface marker expression was not determined simultaneously. Furthermore, our results indicate that adipogenic differentiation potential decreases upon replicative senescence whereas the propensity for osteogenic differentiation increased in higher passages. These observations are based on either fat droplet formation or calcium phosphate deposition and they would be further strengthened by analysis of adipocyte or osteocyte specific gene expression. Our results are in line with similar studies from other groups that demonstrate functional implications on differentiation potential [10], [11], [34], [37]. Thus, *in vitro* expansion attenuates the parameters that are commonly used to define MSC.

To gain insight into the molecular characteristics of replicative senescence, we analyzed changes in mRNA expression profiles of MSC. So far, comparison of expression patterns in early and late passages has only been investigated in other cell types [38], [39]. Differential expression of 10 selected genes was highly consistent in six donor samples as well as in MSC that were isolated under different culture conditions. We have previously demonstrated that variation between these culture conditions has a tremendous impact on gene expression and protein expression profiles [20], [40]. The consistence of senescence associated differential gene expression in different donor samples and under different culture conditions indicates that senescence of MSC preparations follows a common molecular program.

Ontogenetically, it was striking to see, that genes involved in cell cycle, DNA replication and mitosis were significantly less expressed in senescent cells. This further strengthens the hypothesis that senescence follows a fixed program where genes involved in the proliferation machinery are down-regulated. The causal factors that might mediate this process are yet unknown but they might include successive changes in the epigenetic state [13]. On the other hand, these findings do not rule out the possibility that the accumulation of cellular defects (e.g. oxidative stress, telomere loss or DNA damage) activates a specific program for senescence.

Surprisingly, the most significant over-representation of up-regulated and down-regulated genes was at the same locus on chromosome 4q21. Other authors have previously indicated that cell senescence-related genes are localized on human chromosome 4 as introduction of normal human chromosome 4 into three immortal cell lines resulted in loss of proliferation and reversal of the immortal phenotype [41]. The candidate interval has been further specified to a region between 4q22-q23 by analysis of microsatellite markers on the introduced chromosome [42]. Furthermore, a locus on chromosome 4 has been identified by genome-wide scans for linkage of human exceptional longevity and these linkage results indicate the existence of one or more genes that exert a substantial influence on the ability to achieve exceptionally old age [43], [44]. Thus, our differential gene expression analysis provides further evidence that this chromosomal region plays a central role in senescence and aging.

We have demonstrated for the first time that changes of senescence-associated gene expression are not restricted to senescent passages, but increase continuously during *in vitro* expansion. This is in line with the continuous changes in morphology and loss of differentiation potential. These changes might either be based on the subsequent accumulation of growth arrested senescent cells or on the continuous up-regulation of a program involved in senescence. Either way, our results indicate also on a molecular basis, that replicative senescence is a continuous process starting at time of initiation of *in vitro* cultures.

We have analyzed the role of miRNA expression in the molecular determination of cellular senescence. miRNAs constitute a group of endogenous small, non coding RNAs of approximately 22 nucleotides in length that exert a post-transcriptional effect on gene expression [45], [46]. These mature miRNA molecules, cleaved from 70–100 nucleotide hairpin pre-miRNA precursors [47], are expressed in a tissue- and cell-type specific manner and play essential roles in development. For the nematode worm *Caenorhabditis elegans* it was demonstrated that over-expression of lin-4 led to extended life span, and an overall age-related decline in miRNA expression was observed [48], [49]. Other authors reported unchanged miRNA expression patterns in the aging lung of mice [50]. Currently, 556 different human miRNAs are listed in the miRBase registry (miRBase v10, http://microrna.sanger.ac.uk/) and 322 of these are represented on our miCHIP (miRBase v8.0) [29]. We have demonstrated for the first time that miRNAs (hsa-mir-371, hsa-mir-369-5P, hsa-mir-29c, hsa-mir-499 and hsa-let-7f) are up-regulated upon replicative senescence. For target analysis we compared the results from the three miRNA target databases miRBase (http://microrna.sanger.ac.uk/), mirTar (http://mirtar.mbc.nctu.edu.tw/index.html) and Tarbase (http://www.diana.pcbi.upenn.edu/cgi-bin/search.cgi). Despite the fact that each of these miRNAs has a multitude of predicted targets hardly any of these are so far validated. Interestingly it was shown that hsa-mir-29c directly targets DNA-methyl transferase 3A (DNMT3A) and 3B (DNMT3B) in lung cancer tissue [51]. In addition hsa-mir-371 is predicted to target DNMT3A (miRTar), while DNA-methyl transferase 2 (DNMT2) belongs to the predicted targets of hsa-miR-499 (miRTar). Hence, it might be speculated that senescence associated up-regulation of these miRNAs results in changes in the methylation pattern. These epigenetic modifications have been postulated to play a role in senescence [13], [52].

Replicative senescence does not necessarily represent an inevitable fate for all cells, because cellular senescence is not observed in primitive organisms such as sponges or corrals as well as in our germline cells. Somatic cells that have divided many times will have accumulated DNA mutations and would therefore be in danger of becoming cancerous if cell division continued. This implies that in somatic cells replicative senescence might be a purposeful program to protect the organisms rather than a cell culture artifact. Our results indicate that replicative senescence is associated with very reproducible changes in gene expression of MSC from different donors as well as under different culture conditions. Genes involved in DNA replication and repair are successively down-regulated. The final existence of a senescence associated program remains to be proven and specific factors that would activate or regulate such a program are yet unknown. However, the parallel nature of the molecular changes described in this study further indicates that senescence represents a somehow organized process. It is also striking, that these changes increase successively with every passage in a continuous fashion and that they are not restricted to the end of long-term *in vitro* culture. The characteristics of MSC change almost undetectably from the beginning of *in vitro* culture. Thus, future quality standards will have to include the senescence state of MSC preparations. Gene expression profiling and miRNA analysis as presented in this study pave the way for molecular characterization of cell preparations including their state of senescence.

## Materials and Methods

### Isolation of MSC

Human bone marrow (BM) samples were taken after written consent using guidelines approved by the Ethic Committee on the Use of Human Subjects at the University of Heidelberg. In this study, we have used specimen from 8 healthy donors. The mononuclear cell (MNC) fraction was isolated by Biocoll density gradient centrifugation (d = 1.077 g/cm^3^; Biochrom, Berlin, Germany). MNC were plated at a density of 10^5^ cells/cm^2^ in tissue culture flasks (Nunc, Wiesbaden, Germany) under two standardized culture conditions as described in our previous work (MSC^M1^ and MSC^M2^) [20], [40].

Culture medium M1 has been described by M. Reyes and colleagues [1]: It consists of 58% Dulbecco's Modified Eagles Medium-Low Glucose (DMEM-LG, Cambrex, Apen, Germany) and 40% MCDB201 (Sigma, Deisenhofen, Germany), 2% FCS (HyClone, Bonn, Germany), supplemented with 2 mM L-Glutamine, 100 U/ml Pen/Strep (Cambrex), 1% insulin transferrin selenium, 1% linoleic acid bovine serum albumin, 10 nM dexamethasone, 0.1 mM L-ascorbic-acid-2-phosphate (Sigma, Hamburg, Germany), PDGF-bb and EGF (10ng/ml each, R&D Systems, Wiesbaden, Germany). Tissue culture flasks were coated with 10 ng/ml fibronectin (Sigma) before use.

Culture medium M2 is the commercially available Poietics Human Mesenchymal Stem Cell Medium (PT-3001, Cambrex). MSC^M2^ were expanded without fibronectin coating following the manufacturer's instructions.

### Expansion and sampling of MSC

MSC were cultured at 37°C in a humidified atmosphere containing 5% carbon dioxide with medium changes twice a week. After 7–10 days, initial colonies were photo-documented, trypsinized and re-plated in a new culture flask (passage 1, P1). Upon sub-confluent growth at a density of 70%, cells were harvested according to the standardized protocol and re-plated at a density of 10^4^ cells/cm^2^. Photo documentation and cell counting by using a counting chamber was performed at every passage. Cumulative population doublings were calculated as previously described [35]. As cell numbers were first determined at P1, the cumulative doubling number was first calculated for P2. From P2 onward, there were enough cells for simultaneous expansion of one fraction and harvesting another fraction for subsequent analyses: 10^6^ cells were lysed in TRIzol and stored at −80°C for RNA isolation, 10^6^ cells were pelleted and stored at −80°C for DNA preparation and the remaining cells were cryopreserved for immunophenotyping and *in vitro* differentiation.

### Immunophenotypic analysis

Cryopreserved samples of different MSC passages of the same donor were simultaneously taken into culture at density of 2×10^4^ cells/cm^2^ and labeled with the following anti-human antibodies: CD13-allophycocyanin (APC, clone WM15, Becton Dickinson [BD], San Jose, USA), CD29-fluorescein isothiocyanate (FITC, MEM-101a, Abcam, Cambridge, UK), CD34-phycoerythrin (PE, 8G12, BD), CD44-PE (g44-26, BD), CD45-FITC (2D1, BD), CD73-PE (AD2, BD), CD90-PE (G7, BD), CD105-PE (MHCD10504, BD), CD146-PE (P1H12, BD), CD166-PE (3A6, BD), CD184-PE (12G5, BD). Dead cells were discriminated as PI positive. More than 5×10^4^ labeled cells were acquired and analyzed using an upgraded 5-color FACScan flow cytometry system (Cytek Development Inc., Fremont, USA) running CellQuest 3.3 software (BD).

### *In vitro* differentiation

*In vitro* differentiation was simultaneously analyzed in different cryopreserved MSC passages of the same donor. To induce osteogenic differentiation, cells were re-plated at 2×10^4^ cells/cm^2^ and cultured for three weeks in DMEM with 10% FCS (Invitrogen), 10 mM β-glycerophosphate, 10^−7^ M dexamethasone, and 0.2 mM ascorbic acid and with medium changes every 3 to 4 days as previously described [1], [53]. After 21 days, cells were analyzed by either von Kossa or Alizarin red staining. Alizarin red staining was semiquantitatively analyzed at λ595 nm using a plate reader (Bio-TEK-instruments Inc., Winooski, VT, USA). For adipogenic differentiation, cells were plated at 2×10^4^ cells/cm^2^ and cultured in DMEM with 10% FCS, 0.5 mM isobutyl-methylxanthine (IBMX), 1 µM dexamethasone, 10 µM insulin, 200 µM indomethacin, and Oil Red-O staining was performed after 21 days [19] and analyzed semiquantitatively at λ595 nm as described above. Chondrogenic differentiation was achieved through culturing of a pellet of 2.2×10^5^ cells in differentiation medium for three weeks with subsequent assessment of acid mucopolysaccharides by 1% Alcian blue (Chroma, Köngen, Germany) for 10–30 min. [54].

### Senescence associated β-galactosidase staining

Expression of pH-dependent senescence associated β-galactosidase (SA-β-gal) activity was analyzed simultaneously in different passages of MSC using the SA-β-gal staining kit (Cell Signaling Technology, Boston, MA) [24].

### RNA isolation

Total RNA was isolated using TRIzol reagent (Invitrogen, Paisley, Scotland) according to the manufacturer's instructions. RNA quality was controlled using the RNA 6000 Pico LabChip kit (Agilent, Waldbronn, Germany) and quantified with a NanoDrop ND-1000 Spectrophotometer (Nanodrop Technologies, Wilmington, USA).

### Microarray analysis

Two µg total RNA was amplified with GeneChip one-cycle target labeling kit (Affymetrix, High Wycombe, United Kingdom) according to the manufacturer's instructions. Quality of amplified RNA was controlled by LabChip technology (only the sample of donor 1 P9 could repeatedly not be amplified). GeneChip Human Genome U133_Plus_2.0 (Affymetrix) were hybridized with 15 µg amplified RNA, washed with a fluidics station 450 (Affymetrix), and scanned with GeneChip scanner 3000 (Affymetrix). The complete microarray data have been deposited in NCBIs Gene Expression Omnibus (GEO, http://www.ncbi.nlm.nih.gov/geo/) and are accessible through GEO Series accession number GSE9593. For subsequent analyses, only those probe sets were considered that were detectable in at least 10 of the 13 hybridizations (19,448 ESTs). Microarray data were normalized to the median and log_2_ratios were calculated *versus* P2 of the corresponding donor sample. Genes that were more than 2-fold up- or down- regulated in median of all three donor samples were further classified by GeneOntology analysis using GoMiner software (http://discover.nci.nih.gov/gominer/) and representation in functional categories was analyzed by Fischer's Exact p-value test (*P*<0.0001). Lists of regulated genes were assembled using Significant Analysis of Microarrays (SAM) [55]. Unsupervised hierarchical cluster analysis was performed by Euclidean distance (average linkage clustering) using the MultiExperiment Viewer (MeV, TM4) [56]. Chromosomal distribution of differentially regulated genes was analyzed by Chromosomal Co-Localization probability calculator (ChroCoLoc) [57].

### Quantitative real-time PCR analysis

Quantification of mRNA expression for candidate genes was performed by real-time quantitative PCR (QRT-PCR) using the ABI PRISM® 7700HT Sequence Detection System Instrument (Applied Biosystems, Applera Deutschland GmbH, Darmstadt, Germany). Total RNA was reverse transcribed by using the high capacity cDNA reverse transcription kit (Applied Biosystems). Primers were obtained from Biospring (Frankfurt, Germany) (Table 2). QRT-PCR reactions were performed with the power SYBR® green PCR master mix in a MicroAmp optical 96-well reaction plate with a ABI PRISM® 7700HT sequence detector (Applied Biosystems) according to the manufacturer's instructions. Relative gene expression levels were normalized to GAPDH expression, which was used as a housekeeping gene.

**Table 2:** Primer sequences.

Gen	amplicon length (bp)	Forward Primer	Reverse Primer
GAPDH	142	TTCGTCATGGGTGTGAACCA	CTGTGGTCATGAGTCCTTCCA
GPNMB	213	GCATGGTCAGAGGACAGTGA	GAGTTGAGGCCCAAGTGTCA
RAMP	220	CAGGTTCAGTCAAGGGAGACA	TCCACTTCCCAGTCCTCAGA
PERP	187	GTTCCAGATCATCTCCCTGGTA	GCCCAGAAGGTCATCTTCGTA
LY96	261	TCTGCAACTCATCCGATGCA	CTCCCTTCAGAGCTCTGCAA
STAT1	210	GGGTCCTCTCATCGTTACTGA	GGTGGAGTCAGGAAGAAGGA
PRNP	214	GCCTATTACCAGAGAGGATCGA	CCTATCCGGGACAAAGAGAGA
HAS1	180	CTGGGTCAGCTTCCTAAGCA	GTACAGTGGGTACCCAGGA
ID1	157	GGTAAACGTGCTGCTCTACGA	GGATTCCGAGTTCAGCTCCAA
RARRES1	243	ACTTGTACACGGCTCATCGA	CCACTGCCTCACACTAGTGA
TNFSF11	219	CAGAGCGCAGATGGATCCTA	TCTGCTCTGATGTGCTGTGA

### miRNA profiling

Total RNA (3 µg) was labeled with a Cy3-conjugated RNA linker (Biospring, Frankfurt, Germany) and hybridized to miCHIP as previously described [29], [58]. miCHIP is based on locked nucleic acid (LNA) technology, whereby LNA-modified, Tm-normalized miRCURY capture probes (Exiqon, Vedbaek, Denmark) designed to target 322 unique human miRNAs (miRbase v8.0, Wellcome Trust Sanger Institute, http://microrna.sanger.ac.uk/) were printed onto Codelink slides (GE Healthcare, Chalfont St Giles, United Kingdom). Array images were generated by using the Genepix 4200AL laser scanner (Molecular Devices, Sunnyvale, USA) in batches using the Genepix auto PMT (Photo Multiplayer) algorithm, with pixel saturation tolerance set to 0.2%. Tiff images generated by the Genepix 4200AL laser scanner were processed by the Genepix 6 microarray analysis software (Molecular Devices, Sunnyvale, USA). Artifact-associated spots were eliminated both by software- and visual-guided flags. Signal intensities were measured according to the local background subtraction method. All probes were spotted in quadruplicate and the median signal intensity of these was calculated. This dataset has also been deposited in GEO and are accessible through the series accession number GSE9664. MiCHIP data were median normalized and the log_2_ratios of P2 *versus* the corresponding senescent passage was determined. For subsequent analyses, only those probe sets were considered that were detectable in at least 10 of the 14 hybridizations (264 miRNAs). SAM analysis and hierarchical cluster analysis was applied using the same parameters as described for mRNA expression data [55], [56].

### RT-PCR analysis of miRNA expression

Expression of hsa-mir-29c, hsa-mir-369-5p and hsa-let-7f was further analyzed using miRNA TaqMan assays according to the manufacture's instructions (Applied Biosystems, Foster City, USA). Ten ng of total RNA was used for each reverse transcription. QRT experiments were performed by using the ABI 7500 real time PCR system (Applied Biosystems). Relative miRNA expression levels were determined in relation to RNU24 abundance.
